# Maternal death after oocyte donation at high maternal age: case report

**DOI:** 10.1186/1742-4755-5-12

**Published:** 2008-12-30

**Authors:** Joke M Schutte, Nico WE Schuitemaker, Eric AP Steegers, Jos van Roosmalen, the Dutch Maternal Mortality Committee

**Affiliations:** 1 University Medical Center Groningen, Groningen, The Netherlands; 2 Diaconessen Hospital Utrecht, Utrecht, The Netherlands; 3 Erasmus MC, University Medical Center Rotterdam, Rotterdam, The Netherlands; 4 Leiden University Medical Center, Leiden, The Netherlands; 5 VU University Medical Center Amsterdam, Amsterdam, The Netherlands

## Abstract

**Background:**

The percentage of women giving birth after the age of 35 increased in many western countries. The number of women remaining childless also increased, mostly due to aging oocytes. The method of oocyte donation offers the possibility for infertile older women to become pregnant. Gestation after oocyte-donation-IVF, however, is not without risks for the mother, especially at advanced age.

**Case presentation:**

An infertile woman went abroad for oocyte-donation-IVF, since this treatment is not offered in The Netherlands after the age of 45. The first oocyte donation treatment resulted in multiple gestation, but was ended by induced abortion: the woman could not cope with the idea of being pregnant with twins. During the second pregnancy after oocyte donation, at the age of 50, she was mentally more stable. The pregnancy, again a multiple gestation, was uneventful until delivery. Immediately after delivery the woman had hypertension with nausea and vomiting. A few hours later she had an eclamptic fit. HELLP-syndrome was diagnosed. She died due to cerebral haemorrhage.

**Conclusion:**

In The Netherlands, the age limit for women receiving donor oocytes is 45 years and commercial oocyte donation is forbidden by law. In other countries there is no age limit, the reason why some women are going abroad to receive the treatment of their choice.

Advanced age, IVF and twin pregnancy are all risk factors for pre-eclampsia, the leading cause of maternal death in The Netherlands.

Patient autonomy is an important ethical principle, but doctors are also bound to the principle of 'not doing harm', and do have the right to refuse medical treatment such as IVF-treatment. The discussion whether women above 50 should have children is still not closed. If the decision is made to offer this treatment to a woman at advanced age, the doctor should counsel her intensively about the risks before treatment is started.

## Background

In the Netherlands the proportion of women giving birth after the age of 35 increased from 5.2% in 1980 to 20% in 2003 [1]. This has also been reported in other countries [2].

The number of women remaining childless also increased, mostly due to aging oocytes [3]. The method of oocyte donation from younger women and in vitro fertilization (IVF), offers the possibility for infertile older women to become pregnant. However, this is not without risks for the mother [4].

In The Netherlands, the age limit for women receiving donor oocytes is 45 years [5]. In many other countries the maximum age is higher and sometimes even without limits. Also, in The Netherlands, commercial oocyte donation is forbidden by law (so called embryo law, implemented in 2002), therefore, some women are going abroad to receive the treatment of their choice.

In this short communication we present a case of maternal death, illustrating that the application of new technologies of assisted reproduction in high income countries creates a new group of women at risk of maternal death.

## Case presentation

The woman got pregnant for the first time at the age of 36 but had a miscarriage twice. When she was 38 years old, she had two induced abortions. At the age of 41 and 43 years, she got pregnant after ovulation induction, and gave birth to a healthy girl and boy respectively. After this, she suffered from secondary subfertility, but ovulation induction did not result in pregnancy. At the age of 46, after oocyte donation embryotransfer was performed abroad, resulting in a twin pregnancy. However, the woman could not live with the idea of carrying twins. She first opted for termination of pregnancy, but then agreed with selective foeticide in the same clinic. She could still not cope with the pregnancy and opted for abortion of the second twin in the second trimester.

At the age of 49 she again had oocyte donation and IVF in another clinic abroad, resulting in a twin pregnancy. She now seemed to be mentally more stable. Antenatal visits were at a Dutch clinic, and the pregnancy developed uneventful. At a gestational age of 37 weeks her blood pressure had risen from 110/60 to 125/80 mmHg. At 38 weeks labour was induced for elective reasons and the woman delivered a healthy boy and girl, at the age of 50. Shortly after delivery her blood pressure had risen to 170/100 mmHg. She complained of nausea and vomiting. No diagnostic tests, however, were performed and no antihypertensive or anticonvulsant therapy was initiated. Nine hours later she was found unconscious in bed with a tongue bite. Magnesium sulfate was initiated, but she had two more convulsions. Blood results showed signs of HELLP-syndrome. Following the eclamptic fit she was somnolent and disoriented, with a blood pressure of 140/85 mmHg. After 24 hours the CT-scan showed occipital haemorrhage and cerebral oedema. The woman was transferred to the Intensive Care Unit. After a few hours the Glasgow Coma Scale deteriorated and a midline shift was visible on the CT scan. Treatment was stopped because of brain death. Her husband was left behind with four children.

## Discussion

This woman died from eclampsia, after she had a multiple pregnancy at advanced age after oocyte donation IVF. She insisted to have this treatment and being denied IVF in her own country, The Netherlands, she had it performed abroad. The woman had hypertension and pre-eclamptic complaints, but was not managed with antihypertensive medication or magnesium sulphate.

We feel that even the first oocyte donation for this woman could be criticised, but are astonished that doctors did institute such rigorous treatment for the second time, at the age of 49, after the first pregnancy resulting from oocyte donation was chosen to be terminated. The availability of advanced reproductive technology, especially for the wealthier women in society, may create a new, unnecessary group at risk for severe maternal morbidity and mortality in high income countries. Women should be counselled intensively about the risks involved.

IVF, twin pregnancy and advanced age are all risk factors for pre-eclampsia, one of the five major causes of maternal death worldwide, and the leading cause of maternal mortality in The Netherlands.

IVF-pregnancies are associated with more obstetric complications than naturally conceived pregnancies [6]. Källén et al found higher risks of pre-eclampsia (OR 1.63; 95% CI 1.53–1.74), placental abruption (OR 2.17; 95% CI 1.74–2.72) and postpartum haemorrhage (OR 1.4; 9% CI 1.38–1.50) in women being pregnant after IVF-treatment [7]. Venn et al reported an increased risk of maternal mortality in IVF pregnancies: 25.7 per 100.000 pregnancies compared to 10.9 in non-IVF pregnancies [8].

Whether oocyte donation adds an additional risk to the higher risks of obstetric complications in IVF-pregnancies remains controversial, but most studies indicate an additional risk. Söderström-Anttila et al compared obstetric outcome of pregnancy after oocyte donation with pregnancies after standard IVF. In singleton pregnancies they observed 29% (12/41) pregnancy-induced hypertension in the first group versus 12% (8/68) in the other group (*P *< 0.05). Also, the caesarean section rate was higher: 57% (29/51) in the group with oocyte donation versus 37% (36/97) in the standard IVF group [9].

Henne et al found an increased risk of preterm labour, pre-eclampsia and caesarean delivery (after controlling for age and multiple gestations) in recipients of donor oocytes versus women of advanced age with autologous oocytes [10]. Wiggins et al found 26% (6/23) pregnancy-induced hypertension in the group with donor egg IVF as compared to 8% (1/12) in the group with standard IVF. For nulliparous women this difference was even more significant with 37% (13/35) in the donor egg IVF group and 8% (3/37) in the group with standard IVF (OR 7.1; 95% CI 1.4–36.7) [11]. Sauer et al reported in their cohort study in 37.8% (28/74) of pregnancies after oocyte donation obstetrical complications [12]. This was also seen in the cohort of Abdalla, with a high risk of pregnancy induced hypertension and postpartum haemorrhage [13].

Krieg et al compared 71 donor oocyte pregnancies with 108 IVF pregnancies using autologous oocytes at advanced maternal age. They found no differences in incidence of hypertensive disorders, gestational diabetes or mode of delivery after controlling for multifetal gestation, gestational age at delivery and maternal age [14].

Women being pregnant at advanced age have an increased risk for pre-eclampsia and gestational diabetes [15]. Advanced age is also an independent risk factor for caesarean delivery (OR 2.3, 95% CI 1.1–4.8) [16]. Paulson et al reported an incidence of pre-eclampsia of 25% (10/40), being 60% (6/10) in women older than 55 years. The incidence of gestational diabetes was 17.5% (7/40), with an incidence of 40% in women older than 55 years (4/10) [17]. Doctors offering IVF procedures should be aware of the risks named above.

Patient autonomy is an important ethical principle in management, but we feel that there are limits to this. Doctors are also bound to the principle of 'not doing harm' and this principle was at stake in our case [18]. The discussion whether women over the age of 50 should have children is still not closed [2,3]. Landau even reports an increase, rather than a decrease, of human suffering due to the promise of post-menopausal pregnancy with unlimited fertility [19]. Doctors do have the right to refuse to give medical treatment such as IVF-treatment. If it is decided to offer this treatment to a woman at advanced age, she should be counseled intensively about the risks before such treatment is started.

## Conclusion

The discussion whether women at advanced maternal age should become pregnant is still not closed. Doctors, however, have the right to refuse to perform such treatment. Pregnancies at advanced maternal age after oocyte IVF-treatment have increased risks of obstetrical complications. We should be aware of these risks and counsel patients accordingly.

## Consent

Written informed consent was obtained from the husband of the patient for publication of this case report and any accompanying images. A copy of the written consent is available for review by the Editor-in-Chief of this journal.

## Competing interests

The authors declare that they have no competing interests.

## Authors' contributions

All authors have made substantive intellectual contributions to this study. We all contributed to the conception of the article and to the acquisition and analysis of the case. All authors read and approved the final manuscript.
